# Molecular docking and simulation of Zika virus NS3 helicase

**DOI:** 10.1186/s13065-019-0582-y

**Published:** 2019-05-17

**Authors:** Syed Lal Badshah, Nasir Ahmad, Ashfaq Ur Rehman, Khalid Khan, Asad Ullah, Abdulrhman Alsayari, Abdullatif Bin Muhsinah, Yahia N. Mabkhot

**Affiliations:** 1 Department of Chemistry, Islamia College University, Peshawar, Khyber Pakhtunkhwa Pakistan; 2 State Key Laboratory of Microbial Metabolism, Department of Bioinformatics and Biostatistics, Shanghai Jiao Tong University, 800 Dongchuan Road, Shanghai, 200240 China; 3 Department of Pharmacognosy, College of Pharmacy, King Khalid University, Abha, 62529 Saudi Arabia; 4 Department of Pharmaceutical Chemistry, College of Pharamacy, King Khalid University, Abha, 61441 Saudi Arabia

**Keywords:** ZIKV, Microcephaly, Nonstructural protein-3 Helicase, Molecular dynamics simulation, Molecular docking

## Abstract

The Zika virus (ZIKV) has gained attention for the last few years due to the congenital microcephaly and Guillain–Barre Syndrome that resulted in humans. The non-structural protein-3 (NS3) helicase of ZIKV play an important role in viral RNA replication. In this article, we performed hundred nanosecond molecular dynamics simulation and molecular docking of the NS3 helicase of ZIKV with 1,4-benzothiazine derivatives. The root mean square deviation (RMSD) analyses showed the stability of the NS3 helicase. The simulation showed that the flexible and rigid domains of the protein play a crucial role during the RNA replication process. All such domains with ligand binding pockets can be targeted for drug design. The molecular docking showed that the strong hydrogen bonding and arene-cation interactions are responsible for the binding between NS3 and 1,4-benzothiazine derivatives, which provides a new dimension for potent drug design for ZIKV.

**Electronic supplementary material:**

The online version of this article (10.1186/s13065-019-0582-y) contains supplementary material, which is available to authorized users.

## Introduction

Zika virus (ZIKV) is a mosquito-borne flavivirus like yellow fever virus, dengue virus (DENV), West Nile virus (WNV) and chikungunya [1]. It contains a single-stranded positive RNA with a total of 10,794-nucleotides in its genome [2]. The ZIKV transmission occurs through numerous *Aedes spp*. mosquitoes, including *Aedes africanus*, *Aedes luteocephalus, Aedes hensilli, and Aedes aegypti* [3–6]. Initially the ZIKV was observed in rhesus monkey amid observation of yellow fever in the zika woodland in Uganda in the year 1947 and it was initially reported in people in 1952 [7, 8]. Later on in 1954, it was observed in humans with febrile diseases in West Africa [7, 8]. In 2014 several cases of ZIKV were recorded in Indonesia, Micronesia, Thailand, Philippines, French Polynesia and Easter Island in South Pacific [9, 10]. From May, 2015 onward there was a boom in the spread of ZIKV in Brazil and from there it spread to other countries [11, 12].

In early 2016, the World Health Organization declared the ZIKV spread as a international public health emergency due to the presence of congenital neurological disorders like microcephaly, Guillain–Barre syndrome and cranial nerve dysfunction [13]. The exact mechanism of action of ZIKV that how it causes microcephaly is not known but research efforts are in progress to establish the link between ZIKV and microcephaly [10, 14, 15]. Generally, the mosquito borne flavivirus pathogenesis begins with its replication in dendritic cells close to the site of inoculation from where it move to the lymph centers and blood circulation system [16]. After the initial viral attack, the virions can be observed in the blood, while the viral RNA can been seen after eleven days of ailment [17]. The helicases are an omnipresent exceedingly diverse group of proteins that carry out an astonishing variety of functions in cells [18]. These are ATP-ases which depend on nucleic acid and have the potential to unwind DNA or RNA duplex substrates [18]. Because of this unwinding properties, they are significantly involved in every process of cells linked with DNA like replication, repair, transcription, translation, synthesis of ribosomes, RNA maturation and splicing and the procedures related with the nuclear export [18]. Recently a number of structural as well as non-structural proteins from ZIKV that have enzymatic functions are resolved at high resolution through various techniques and they are useful target for drug design [19]. The NS3 helicase is one of the most thoroughly studied antiviral drug target [20, 21]. In NS3 of flaviviruses, the enzymatic activity is coupled with the C-terminal region, namely an RNA helicase (NS3-Hel) concerned with genome replication and RNA synthesis [22, 23]. The NS3-Hel is part of the superfamily helicases, and its inhibition in DENV makes the virus unable to replicate [24, 25]. The two motor domains formed in the helicase part of the NS3 proteins are identical with all other helicases of superfamily 1 and 2 but the third domain is very different from other helicases. The binding site for ATP is located between the two domains while the RNA binds between the third and motor domain [26]. The inchworm like movement of NS3 helicase in a 3′ to 5′ directions along one strand of RNA was observed in detailed mechanistic studies which involved the displacing of the complementary strand in an ATP utilized reaction. The same type of structural and enzymatic studies of DENV helicase suggest that there is no difference between the mechanism of hepacivirus and flavivirus [27, 28]. The non-nucleoside based viral inhibitors are an active area of research and benzothiazine based compounds have been studied against several flaviviruses [21, 29, 30]. They contains a nitrogen and a sulfur atom in their basic nucleus that makes them a good interacting agent inside the active site of an enzyme [30]. Different research groups have shown that benzothiazine are suitable inhibitors of helicases [31, 32]. Currently there are two MD simulation studies available that explore the dynamics of ZIKV NS3 helicase but to get further insight docking novel inhibitors and further simulation studies are required [33]. In this study we targeted the ZIKV NS3 helicase with 1, 4-benzothiazine analogues (Additional file 1: Figure S1) using molecular dynamics simulation and molecular docking methods. The reason for choosing 1,4-benzothiazine based derivatives was that a number of research groups have used 1,4-benzothiazine analogues both computationally and experimentally against different viral helicase enzymes [31, 34]. Therefore, we design these derivatives and used it for molecular docking and simulation studies. We hope that this study will also be vital in the effort to find suitable inhibitors against the spread of this devastating viral disease.

## Methodology

The crystal structure of the NS3 helicase of ZIKV was obtained from Protein Data Bank with the PDB ID 5JRZ [35]. PyMOL software version 1.7 was used for visualization and checking the protein [36]. The ligands were searched from the literature and newly reported compounds were selected for molecular docking [37]. For molecular docking, we used the Molecular Operation Environment (MOE) 2014 docking software program [38]. The prediction of the active site in the protein was carried out with MOE 2014 software, site finder tool which identified the various sites in which one was selected for docking procedure. The removal of water molecules was done from the crystal structure. The addition of missing hydrogen atoms was done, correction of charges and assigning of the correct hybridization state of each residue was done through the preparation program of MOE. The corrected protonation was done using 3D protonate module present in MOE with Generalized Born/Volume Integral (GB/VI) electrostatic function. To explore the potential binding site, the complete structure of the enzyme was used as a receptor. For each ligand, generation of multiple conformations were done by applying a selected torsion angles to all the rotatable bonds in each ligand. For each drug candidate about thirty conformations were generated. For each ligand and the receptor, the accepted conformations were scored by using the London Dock scoring function which calculates the free energy for the binding ligand in a given conformation [39].

The binding affinities were calculated using generalized-Born volume integral/weighted surface area (GBVI/WSA) method present in MOE 2014. Generalized Born interaction energy is the non-bonded interaction energy between the receptor molecule and the ligand. It is composed of Coulomb electrostatic interaction, Van der Waals, and implicit solvent interaction energies. The binding affinity was calculated for each hit after energy minimization, and reported in unit of kcal/mol [40]. The PCA was performed as reported previously [41].

### Molecular dynamics simulations

The crystal structure of NS3 helicase of ZIKV, PDB ID 5JRZ having 1.62 Å resolution was used for atomic coordinates for both Apo and in docked complex with derivative 7 saved as PDB file, which was constructed through SYBYLs-X2.1.1 [42]. For molecular dynamics simulation the AMBER12 software package [43, 44] was utilized adjusting all the parameters. The addition of hydrogen atoms was done through LEaP module of AMBER-12. For the maintenance of neutral condition of the system the counter-ions were utilized. A truncated octahedral box of TIP3P molecules of water was used for the solvation of both the systems. The cutoff distance is kept 8 Å for the determination of pairwise interactions (van der Waals and direct coulombic interactions). In order to compute the long range electrostatic interactions the particle mesh Ewald (PME) method [45] of AMBER12 was used while the ff12SB force field was selected for the finding of the intramolecular interactions. For preparations runs a Langevin thermostat was utilized with friction constant of 1 ps^−1^ while for the production runs a Berendsen thermostat was used [46]. All MD simulations were accelerated using the CUDA version of PMEMD in GPU cores of NVIDIAs Tesla K20. The 500-step steepest descent minimization and 2000-step conjugated gradient minimization were performed for initial minimization with macromolecules frozen in order to avoid further structural clash in the solvated system. The whole system was again passed through the minimization process at the end, keeping 1000-step steepest descent minimization and 19,000-step conjugate gradient minimization. The adjustment of parameters after energy minimization at 400 ps heating up and 200 ps equilibration in the NVT ensemble at 310 K were done before the start of MD simulations in the NPT ensemble at 310 K. In order to carry out a comparison of Apo and complex various simulated trajectories at different nanoseconds were collected at 310 K during the 100 ns simulation timescale.

## Results and discussion

### Molecular docking of 1,4-benzothiazine derivatives

**Fig. 1:**
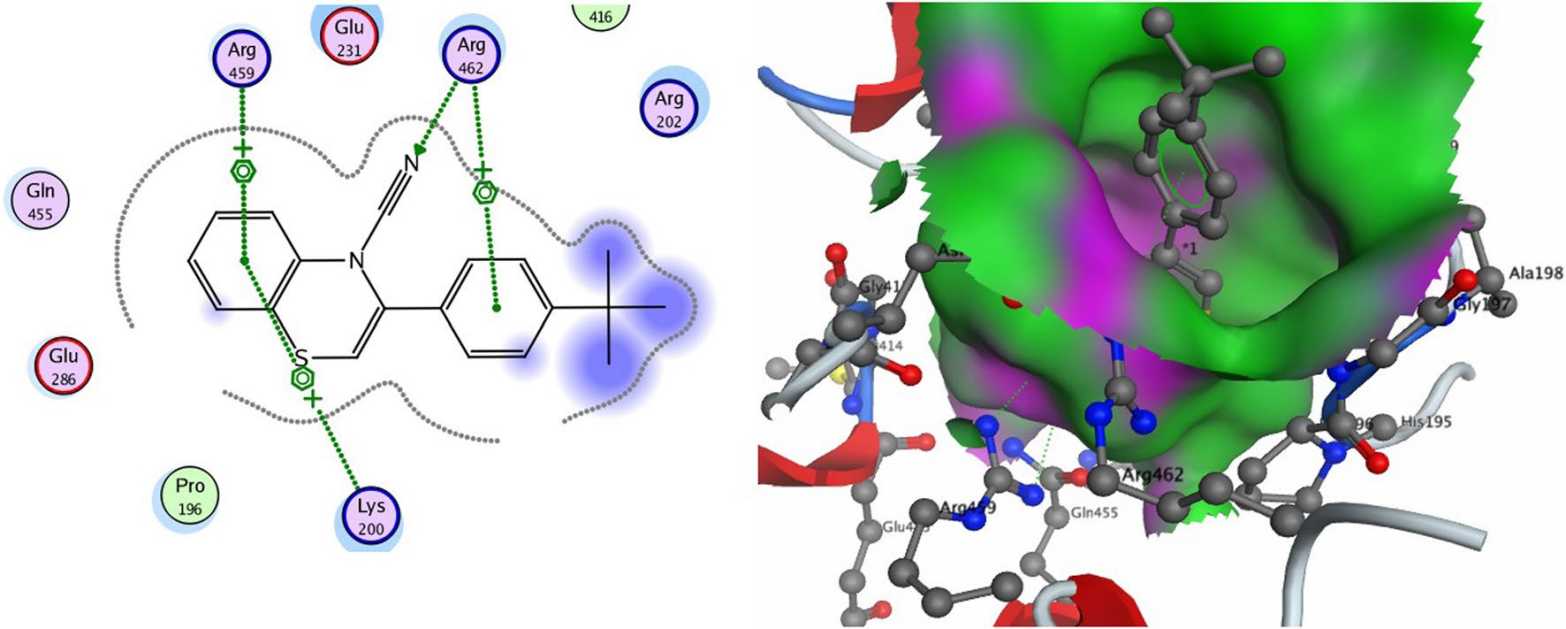
2D and 3D structures of 1,4-benzothiazine analogue 7 with ZIKV NS3 helicase. The inhibitor occupies the ATP binding site and interacts mostly with lysine-200 and arginine-459 and 462 of the active binding pocket

On docking several derivatives of 1,4-benzothiazine with ZIKV NS3 helicase, they bind inside the ATP binding pocket of the enzyme. Most of these inhibitor derivatives interact with the enzyme through hydrogen bonding and cation-arene interaction. In the majority of the derivatives docking with the enzyme, Lys-200, Arg-459 and 462, and Thr-201 were involved in interactions (Fig. 1 and Additional file 1: Figure S2) and Additional file 1: Table S1. The involvements of these residues were also previously recorded by other docking and simulation studies [47, 48].

### Stability of the simulated systems

**Fig. 2:**
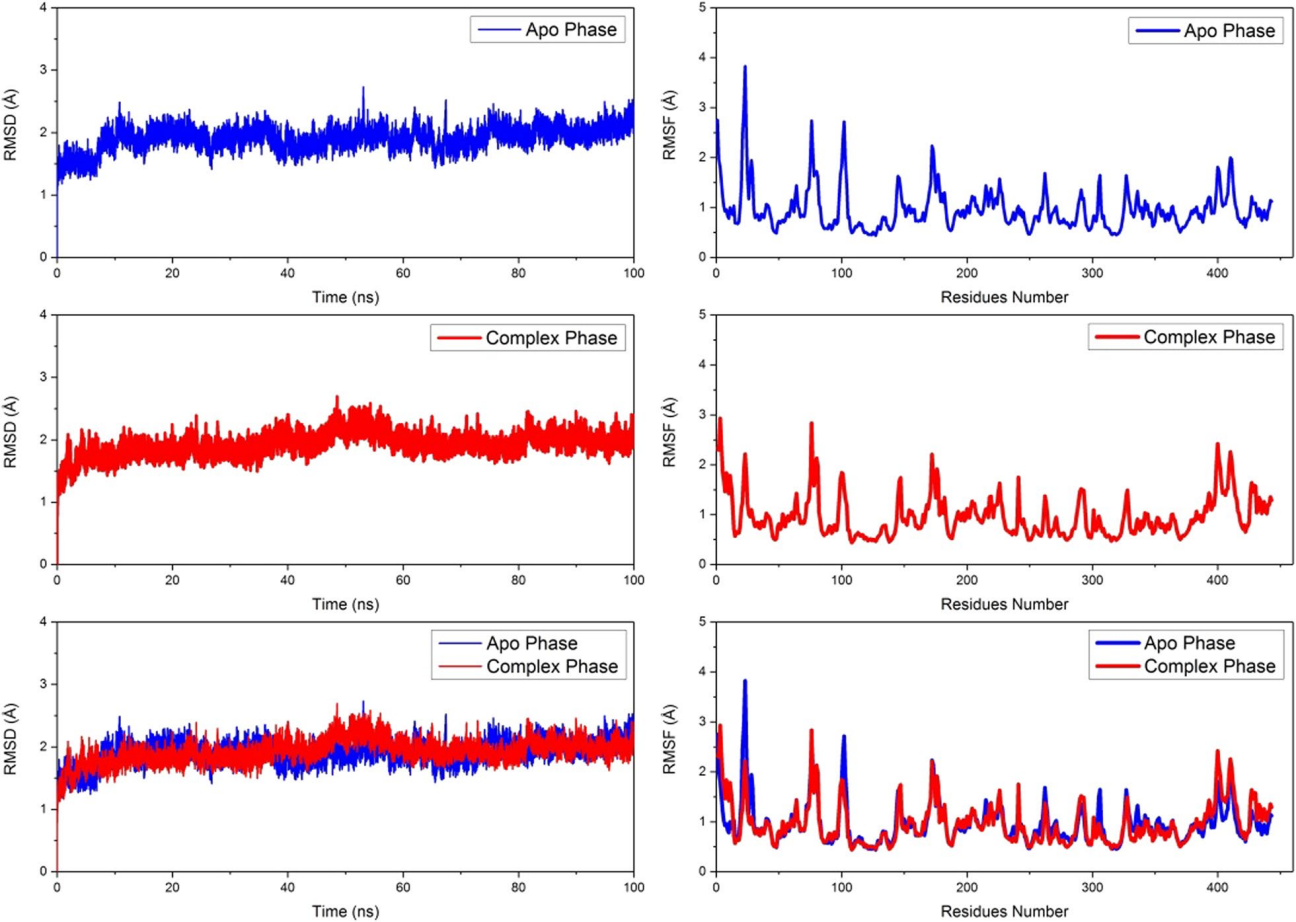
The C-α backbone RMSD of NS3 helicase APO enzyme and in complex with 1,4-benzothiazine derivative 7. The average C-α RMSD was calculated to be 1.8 Å to 1.9 Å, respectively. The right side panel showed the root mean square fluctuations in Apo and in complex with the ligand, while the lower right side panel showed the superposition of the two upper RMSF graphs for better assesment of the residues involved in fluctuations

The molecular dynamics simulation provided us the changes inside the protein under observation during the virtual time and thus provides useful information regarding the protein drug interaction. The longer the time, the better the observations of the different types of interaction and movements of the protein domains. The stability of the system is shown by the changes in the root mean square deviation (RMSD) during the course of simulation time. As shown in Fig. 2, left panel shows the RMSD graphs of the backbone Cα atoms of both Apo and protein in complex with the ligand (1,4-benzothiazine analogue 7). In the start of the simulation there is a 0.5 Å rise in RMSD value for the first few nanosecond, but then it converged for both systems and it is around 1.9 Å. Although there is minor fluctuation in the RMSD value for both the systems, but as a whole both the systems are highly stable during the 100 ns time course (Fig. 2).

### Structural fluctuation of the NS3 protein

**Fig. 3:**
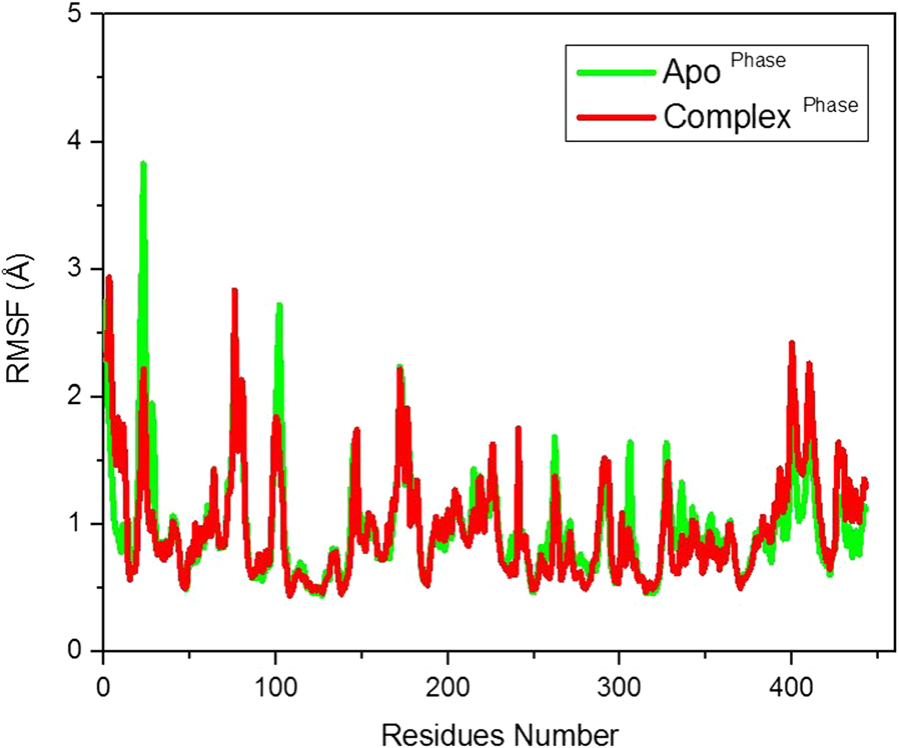
The RMSF of APO protein and its complex with the inhibitor (1,4-benzothiazine derivative 7)

The root mean square fluctuation (RMSF) analysis of the 449 amino acid of the helicase enzyme was calculated to get an insight into the residues that are involved in the interactions with the inhibitor, secondly the most mobile residues and the non-flexible residues are important as they may be further targeted for drug design. It can be observed from Figs. 2 and 3 that the helicase in complex with the inhibitors (1,4-benzothiazine derivative 7) has less fluctuations as compared to the apo form of the protein. The residues number 25-30 are highly flexible with RMSF of 3.7 Å in the apo form, but this value falls to 2 Å in the complex state, showing the interactions between the helicase and the 1,4-benzothiazine derivative 7 in the docked form. In a similar study, Ramharack et al. showed that the NS3 helicase containing more than six hundred residues in complex with its inhibitor NIT008D has more fluctuation then its apo form [47]. While in our simulation of the ZIKV NS3 helicase has on the average same fluctuation in apo and in complex form but variation are there with increase or decrease in RMSF values.

Drug designing involved two opposite views about the drug targets in literature; one is that inhibitors inhibit the most rigid residues and the second view is that inhibitors target highly flexible residues [48]. The rigid residues and flexible regions in enzymes have specified role in various biological reactions [48]. The molecular recognition and catalytic properties are linked with the mobility of specific residues while production of various structures in beta-folds is due to the rigid residues, that are essential for interaction with the substrate and its catalysis.

### Principal component analysis

**Fig. 4:**
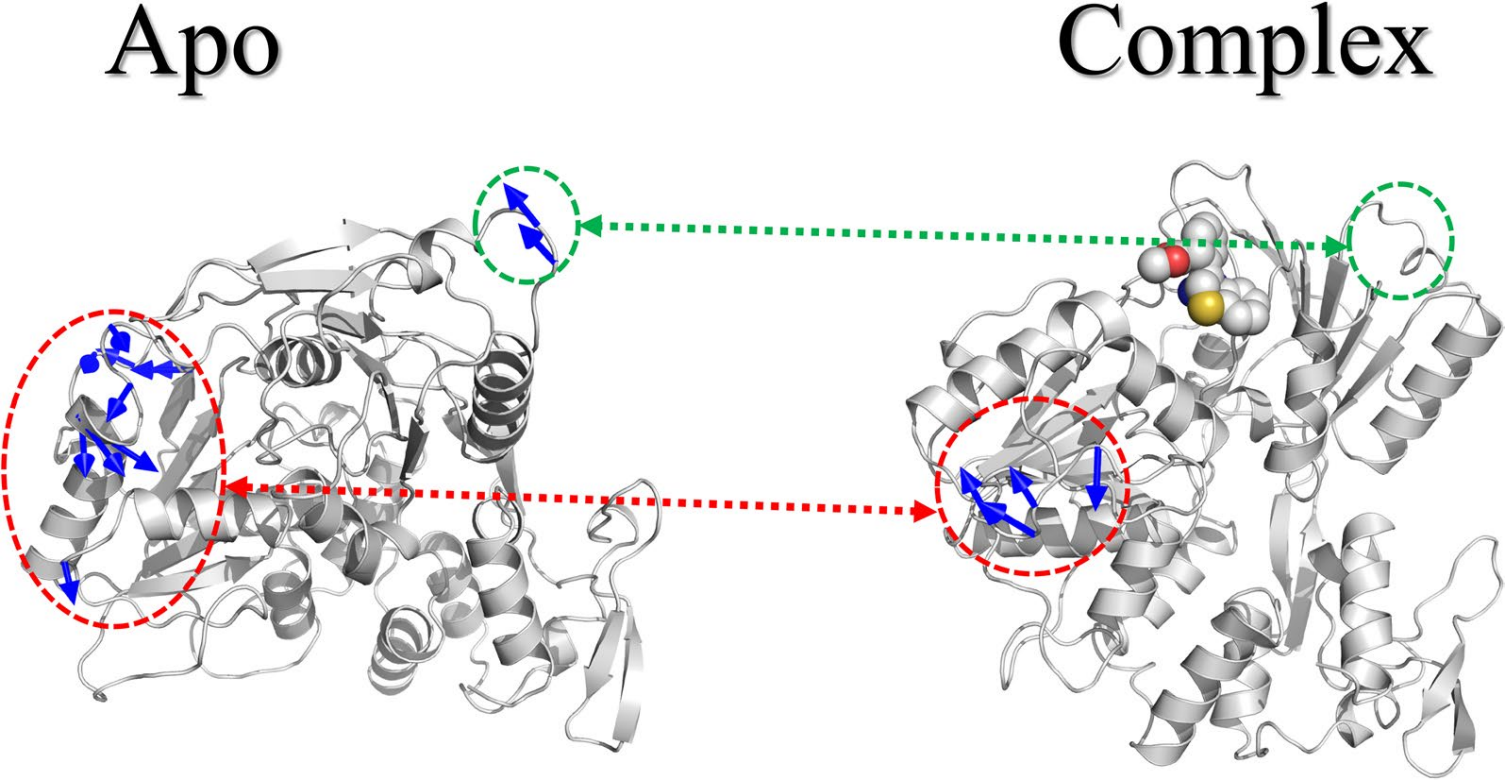
PCA analyses of both Apo and protein in complex with the 1,4-benzothiazine derivative 7 after 100 ns simulations time

The principal component analysis (PCA) was performed for both Apo and complex with the inhibitor (1,4-benzothiazine derivative 7). The simulation results showed that specific movements are present in the domains of the helicase with and without the inhibitor (Fig. 4). In case of Apo it is very clear from the Fig. 4, which in alpha helices and loops of domain II; and loops of domain I there is an anti-clock wise motion which is represented by the blue arrows. It means that there are conformational changes in the enzyme after 100 ns simulations. The motion showed the catalytic importance of that particular region in the enzyme function. In case of the ZIKV helicase in complex with the inhibitor (1,4-benzothiazine derivative 7), a clockwise motion is present in alpha helices and β-sheets of domain II. A similar kind of domain motions were also observed by Ramharack et al. in their simulation studies of ZIKV NS3 helicase [47]. From the PCA analysis it can be concluded that conformational changes are present in both Apo and complex system but their direction and magnitudes are totally different, showing the effects of the inhibitor.

### Interaction analysis

**Fig. 5:**
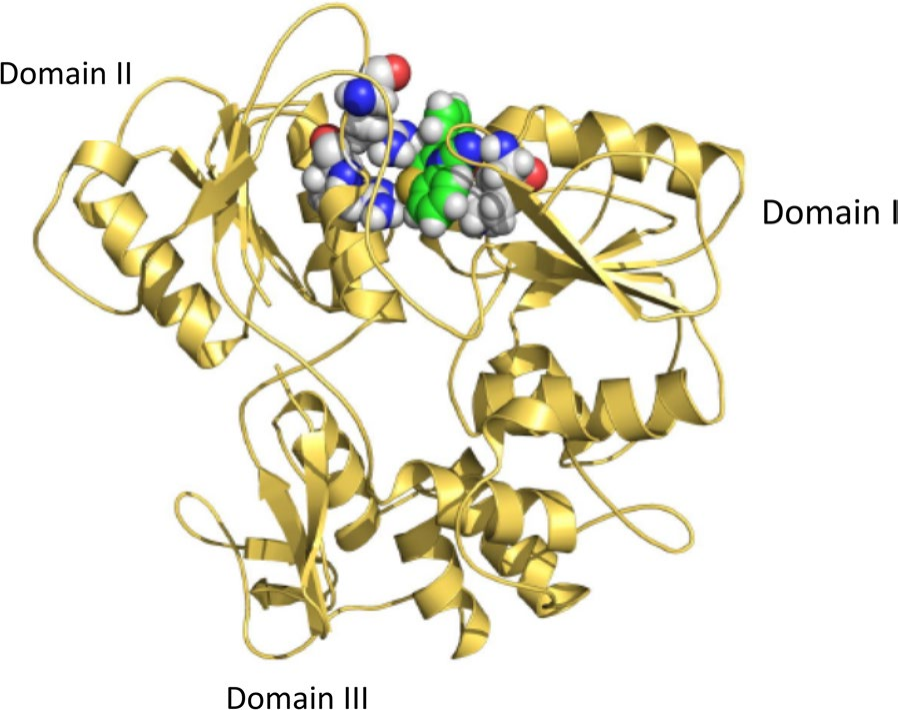
Interaction of 1,4-benzothiazine derivative 7 in the predicted active site of NS3 helicase

The MD simulation of the complex of the NS3 helicase with inhibitor showed that the 1,4-benzothiazine analogue 7 interacted with Lysine200, Arginine462 and Arginine459 in the active site as shown in Fig. 5. In Fig. 5 the colored ball model is derivative 7 while others are important interacting residues of the protein. The docking score of the derivative is − 13.09 kcal/mol with binding free energy of − 12.34 kcal/mol. The good docking score elucidate that the inhibitor is strongly bound in the pocket with a favorable binding energy value. This binding pocket is the same ATP binding site where the inhibitor NITD008, a Flavivirus adenosine analogue bind in the simulation and docking studies performed by Ramharack et al. [47]. The ATP binding site is a hydrophobic pocket and is present between domain I and II [35, 49]. Lysine and arginine residues are playing important role in the interaction and catalysis at this site [35, 49]. Similarly this ATP binding site is the same as in the DENV helicase enzyme [26].

### Superposition of Apo and complex before and after simulation

**Fig. 6:**
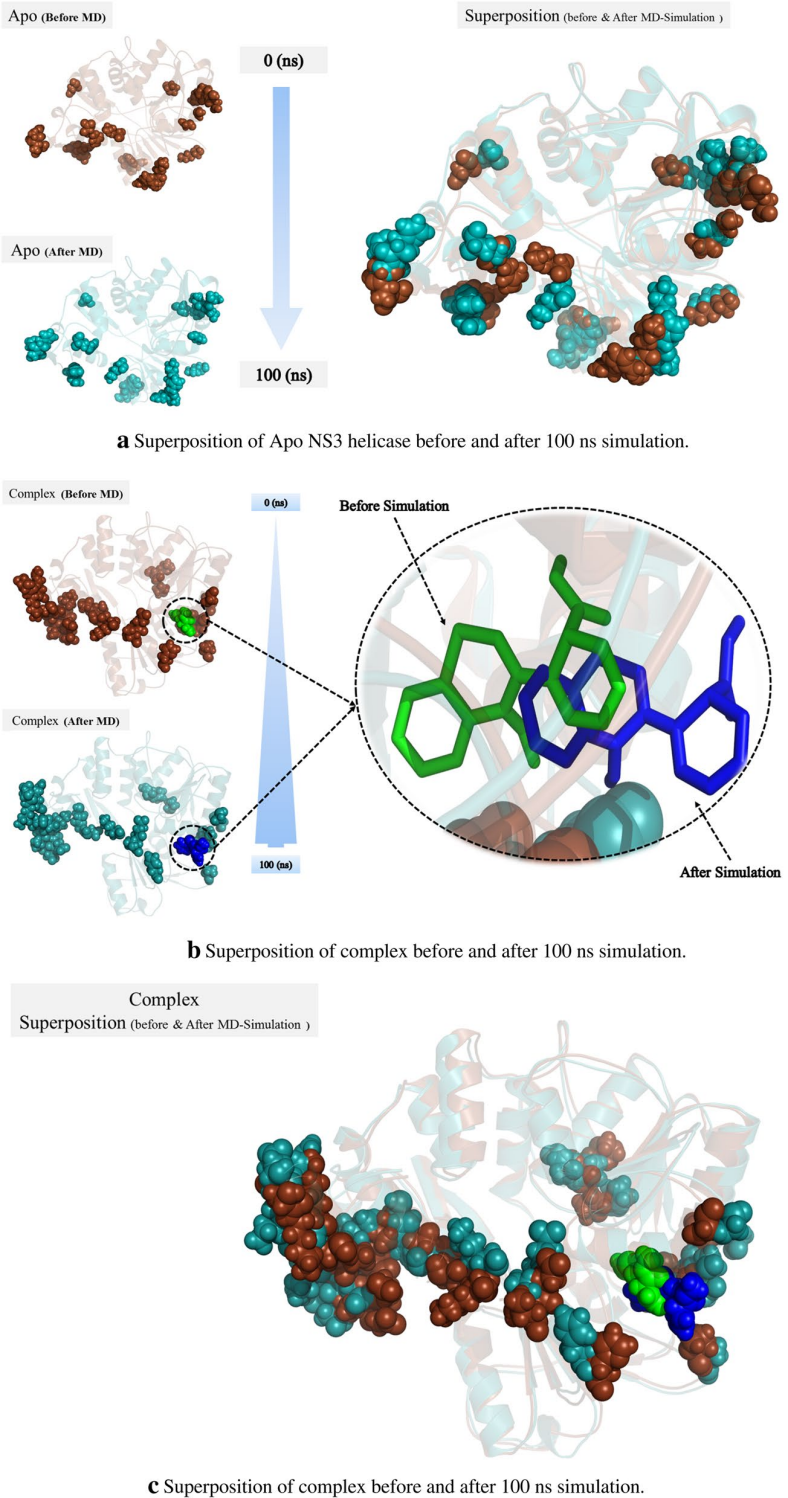
**a** Superposition of Apo NS3 helicase before and after 100 ns simulation. **b** Superposition of complex before and after 100 ns simulation. **c** Superposition of complex before and after 100 ns simulation in a closer view

The superposition of the Apo and complex form were performed before and after simulation of 100 ns as shown in Fig. 6a. The sphere in the model indicated the positions where variations occur with respect to the simulation time. Each sphere showed different degree of motion and also changes in their orientations. The change in position of the spheres showed a displacement toward the center of the axis of the protein from left and right side. This type of motion may be essential for processing the RNA molecule when it enters the helicase and probably a common feature of the flaviviridae NS3 helicases.

In case of the complex form, before the simulation time, the inhibitors was represented by green color while the portions of three domains were represented by dark brown color and after the simulation the ligand was shown by blue color and the protein mobile spheres were represented by cyan color (Fig. 6b, c). When we performed the superposition of the NS3 helicase with the inhibitor, there is a difference in the position of the mobile residues which showed that the inhibitor produced conformational changes when it binds inside the active site of the NS3 helicase which was previously confirmed from RMSF analysis. Secondly, again the amount of displacement and orientation in the spheres is different from one another in the same protein. While an internal motion towards the center of axis from left and right side is also observed in the complex form after the simulation time.

## Conclusion

Helicase enzyme of various viruses is an important drug target and in this study, we reported the utilization of 1-4 benzothiazine derivatives as inhibitor of ZIKV helicase for the docking study. The docking results indicated that these compounds were in interactions mainly with residues **Thr201**, **Lys200, Arg462** and **Arg459** of the NS3 which was reported hotspots by making hydrogen bonds and arene-cation interactions. The 1,4-benzothiazine derivative 7 showed maximum interactions with best docking scores and binding energies while its validation was further carried out with molecular dynamics simulation for 100 ns and the results were found excellent including, RMSD, RMSF values, principal component analysis and superposition expressed the stability of the drug inside the binding pocket. From the above study, we can conclude that this class of compounds can be used as possible novel inhibitors of ZIKV helicase (NS3) and it’s a way forward toward virtual screening and pharmacophore mapping tools to explore the treasures of biological important class of compounds to inhibit the genome replication of ZIKV. The molecular dynamic simulation confirmed that the docked conformation is reliable. Binding energy calculations through the MOE docking program showed that van der Waal interaction and hydrogen bonding provided the most substantial force for the binding of the inhibitor.

## Additional file

**Additional file 1: Figure S1.** The nine different 1,4-benzothiazine derivatives from D-1 to D-9 used in molecular docking against ZIKV NS3 helicase. **Figure S2**. The molecular docking interaction of 1,4-benzothiazine derivatives 1-9 in 2D and 3D. **Table S1.** Docking score and binding energy of 1,4-benzothiazine derivatives.

## Data Availability

Data and material are available on request.
