# Supplementary material for: Molecular docking and simulation of Zika virus NS3 helicase
Source: BMC Chem. 2019 May 17;13(1):67. doi: 10.1186/s13065-019-0582-y (PMC6661806; doi:10.1186/s13065-019-0582-y)
Supplement: Supplementary file 1 — Additional file 1: Figure S1. The nine different 1,4-benzothiazine derivatives from D-1 to D-9 used in molecular docking against ZIKV NS3 helicase. Figure S2. The molecular docking interaction of 1,4-benzothiazine derivatives 1-9 in 2D and 3D. Table S1. Docking score and binding energy of 1,4-benzothiazine derivatives. [file 13065_2019_582_MOESM1_ESM.docx]

| **COMPOUNDS** | **Docking scores (kcal/mol)** | **Binding energy**  **(kcal/mol)** | **Number of Interactions** |
| --- | --- | --- | --- |
| Derivative 1 | -15.26 | -14.34 | 3 |
| Derivative 2 | -14.23 | -14.67 | 3 |
| Derivative 3 | -13.20 | -12.76 | 3 |
| Derivative 4 | -13.66 | -12.09 | 2 |
| Derivative 5 | -12.87 | -11.99 | 3 |
| Derivative 6 | -12.33 | -11.22 | 2 |
| **Derivative 7** | **-13.09** | **-12.34** | **4** |
| Derivative 8 | -12.11 | -11.32 | 2 |
| Derivative 9 | -10.22 | -10.87 | 2 |

Additional Information

**Additional Table S1. Docking score and binding energy of 1,4-benzothiazine derivatives.**





**Additional Figure S1.** The nine different 1,4-benzothiazine derivatives from D-1 to D-9 used in molecular docking against ZIKV NS3 helicase.

| 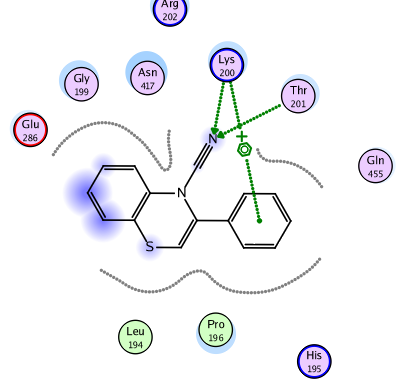 | 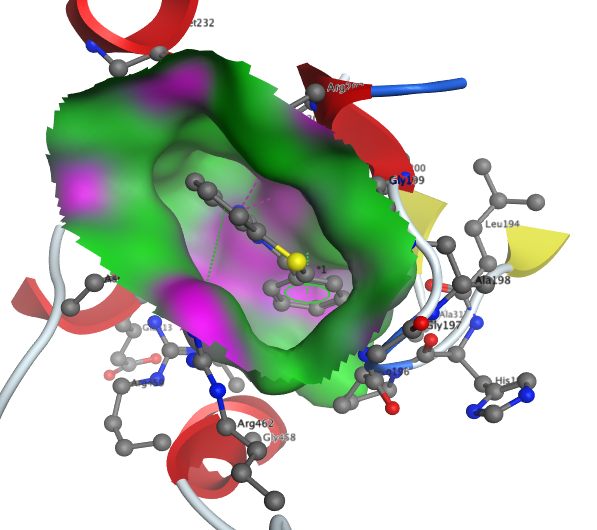 |
| --- | --- |
| 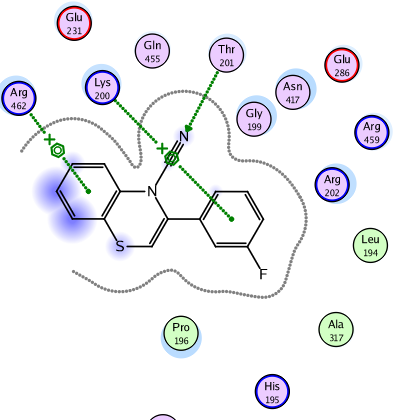 | 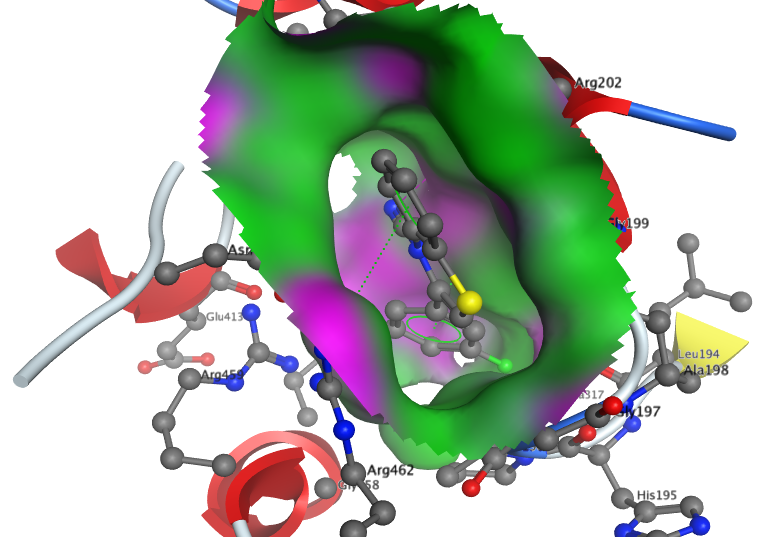 |
| 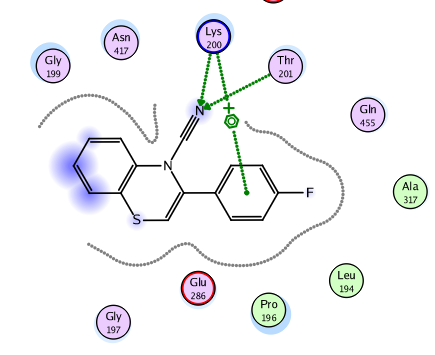 | 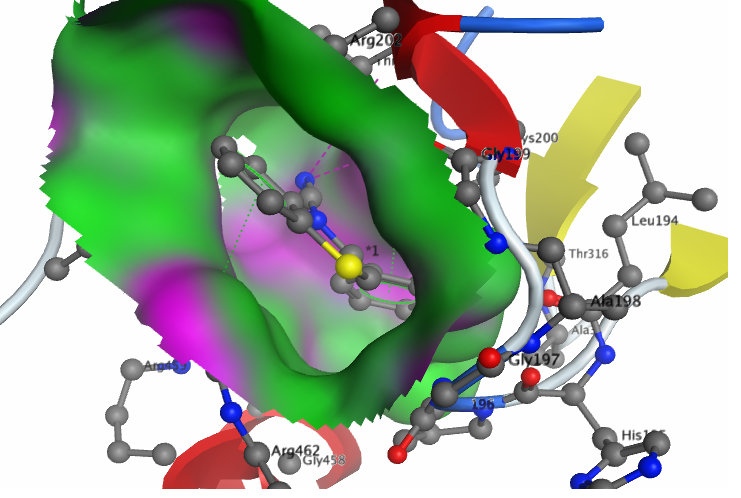 |

| 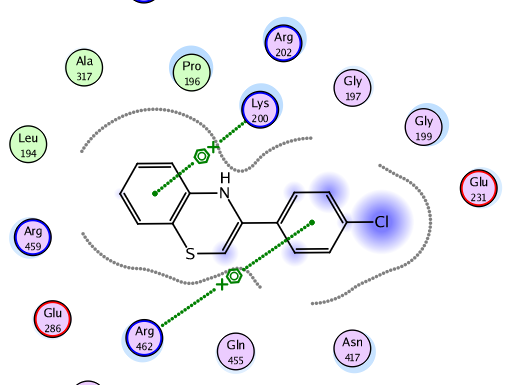 | 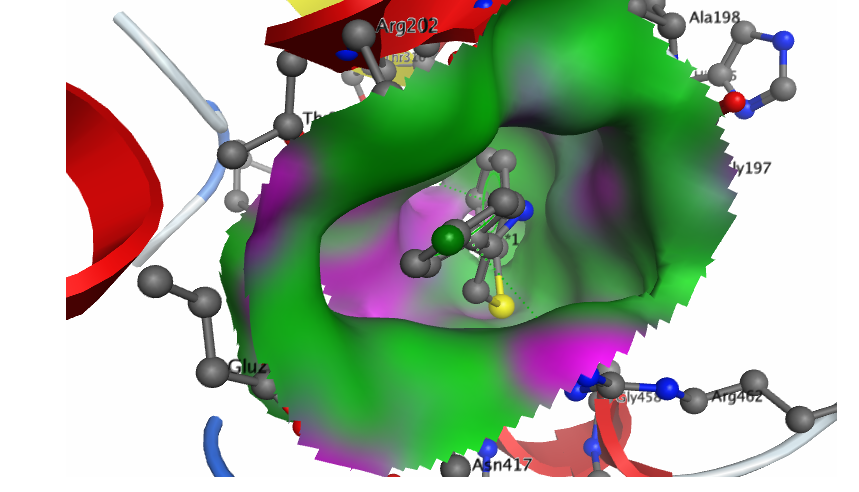 |
| --- | --- |
| 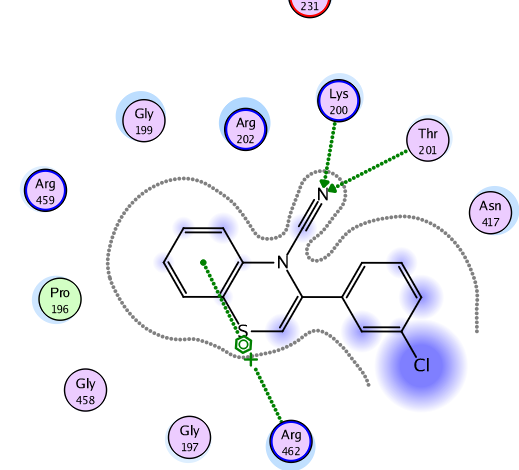 | 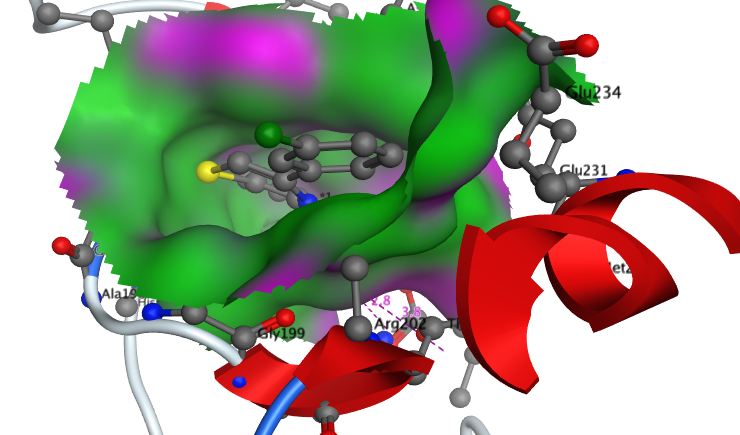 |
| 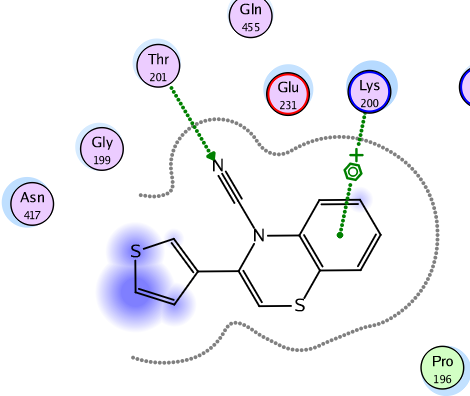 | 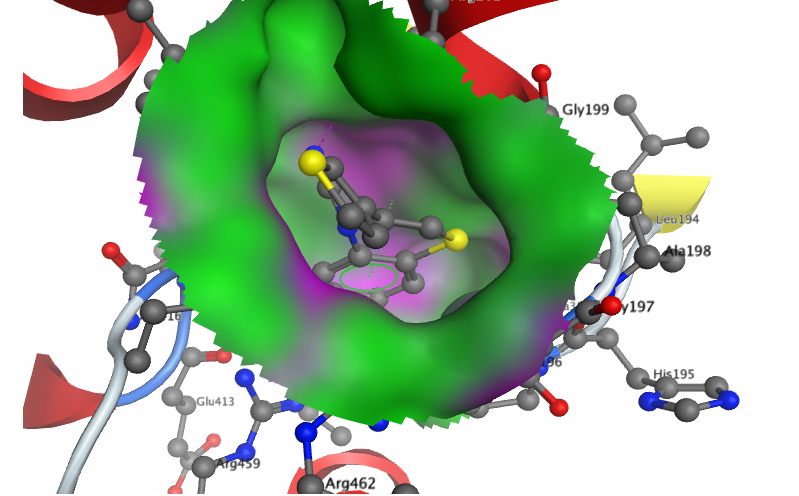 |

| 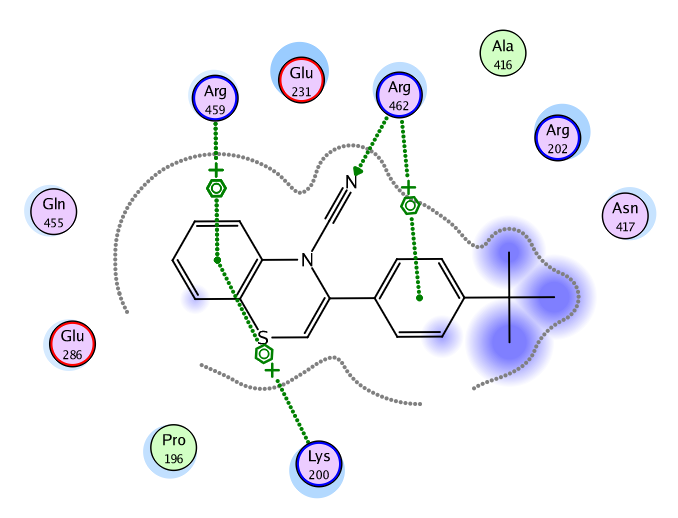 | 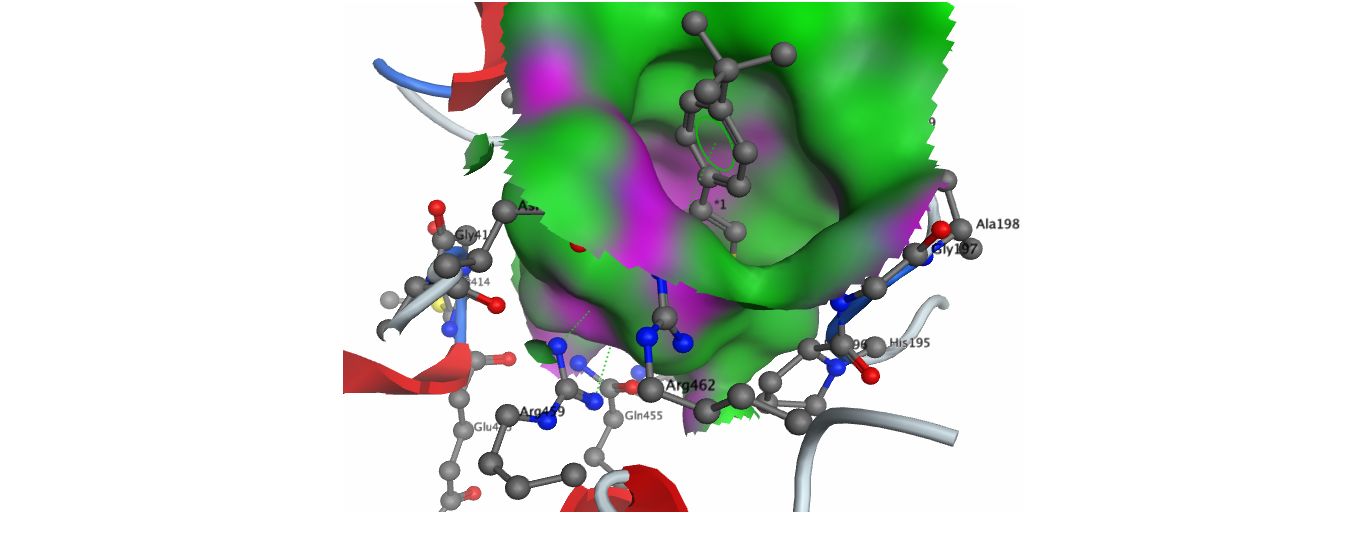 |
| --- | --- |
| 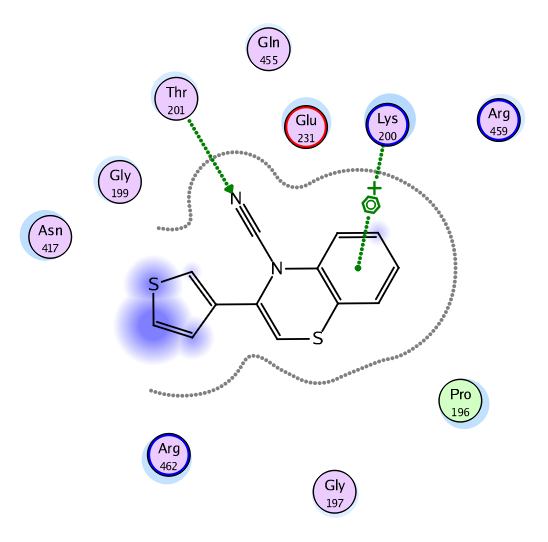 | 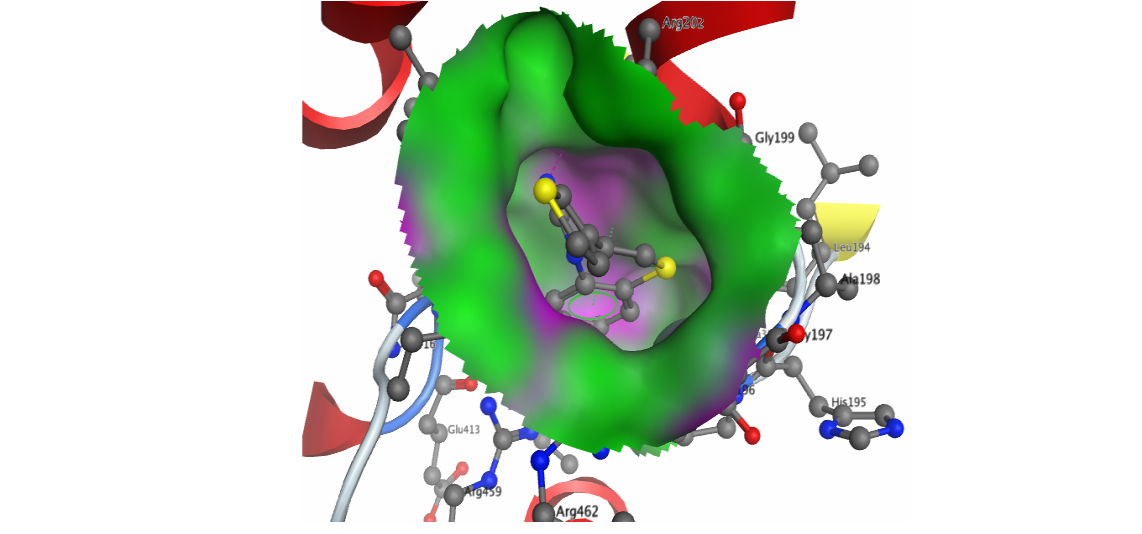 |
| 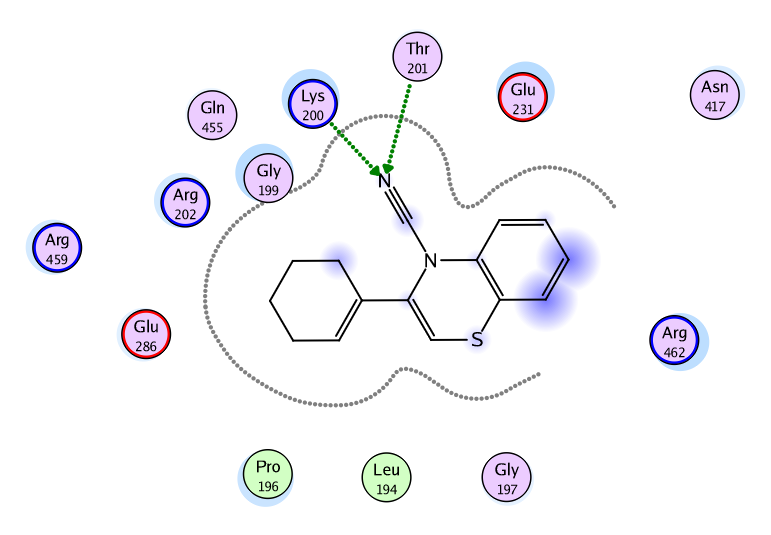 | 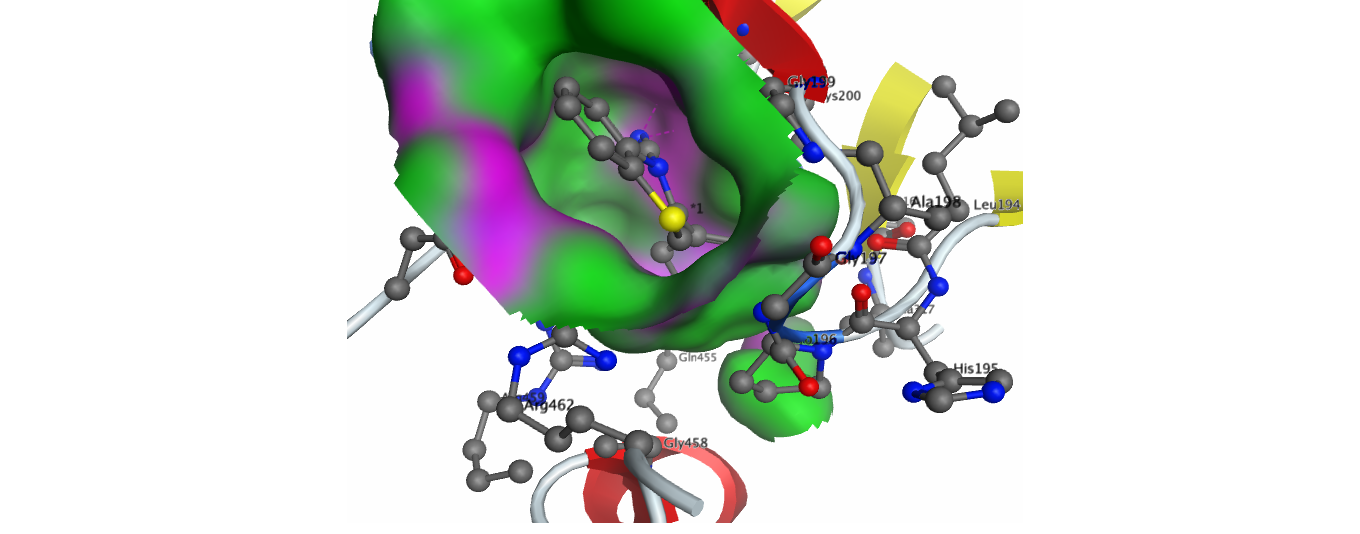 |

**Additional Figure S2**. The molecular docking interaction of 1,4-benzothiazine derivatives 1-9 in 2D and 3D.
